# Experimental evolution of gallium resistance in *Escherichia coli*

**DOI:** 10.1093/emph/eoz025

**Published:** 2019-09-06

**Authors:** Joseph L Graves, Akamu J Ewunkem, Jason Ward, Constance Staley, Misty D Thomas, Kristen L Rhinehardt, Jian Han, Scott H Harrison

**Affiliations:** 1 Department of Nanoengineering, Joint School of Nanoscience & Nanoengineering, North Carolina A&T State University & UNC Greensboro, Greensboro, NC, USA; 2 BEACON Center for the Study of Evolution in Action, Michigan State University, East Lansing, MI, USA; 3 High School Science Teacher, Davie Public High School, Davie, NC, USA; 4 Department of Biology, Bennett College, Greensboro, NC, USA; 5 Department of Biology, North Carolina A&T State University, Greensboro, NC, USA

**Keywords:** experimental evolution, gallium, *Escherichia coli*, genomics

## Abstract

**Background and Objectives:**

Metallic antimicrobial materials are of growing interest due to their potential to control pathogenic and multidrug-resistant bacteria. Yet we do not know if utilizing these materials can lead to genetic adaptations that produce even more dangerous bacterial varieties.

**Methodology:**

Here we utilize experimental evolution to produce strains of *Escherichia coli* K-12 MG1655 resistant to, the iron analog, gallium nitrate (Ga(NO_3_)_3_). Whole genome sequencing was utilized to determine genomic changes associated with gallium resistance. Computational modeling was utilized to propose potential molecular mechanisms of resistance.

**Results:**

By day 10 of evolution, increased gallium resistance was evident in populations cultured in medium containing a sublethal concentration of gallium. Furthermore, these populations showed increased resistance to ionic silver and iron (III), but not iron (II) and no increase in traditional antibiotic resistance compared with controls and the ancestral strain. In contrast, the control populations showed increased resistance to rifampicin relative to the gallium-resistant and ancestral population. Genomic analysis identified hard selective sweeps of mutations in several genes in the gallium (III)-resistant lines including: *fecA* (iron citrate outer membrane transporter), *insl1* (IS30 tranposase) one intergenic mutations *arsC →/→ yhiS*; (arsenate reductase/pseudogene) and in one pseudogene *yedN* ←; (*iapH/yopM* family). Two additional significant intergenic polymorphisms were found at frequencies > 0.500 in *fepD* ←*/→ entS* (iron-enterobactin transporter subunit/enterobactin exporter, iron-regulated) and yfgF ←/→ yfgG (cyclic-di-GMP phosphodiesterase, anaerobic/uncharacterized protein). The control populations displayed mutations in the *rpoB* gene, a gene associated with rifampicin resistance.

**Conclusions:**

This study corroborates recent results observed in experiments utilizing pathogenic *Pseudomonas* strains that also showed that Gram-negative bacteria can rapidly evolve resistance to an atom that mimics an essential micronutrient and shows the pleiotropic consequences associated with this adaptation.

**Lay summary:**

We utilize experimental evolution to produce strains of *Escherichia coli* K-12 MG1655 resistant to, the iron analog, gallium nitrate (Ga(NO_3_)_3_). Whole genome sequencing was utilized to determine genomic changes associated with gallium resistance. Computational modeling was utilized to propose potential molecular mechanisms of resistance.

## INTRODUCTION

The spread of resistance to traditional antibiotics has spurred the search for new antimicrobial substances [1–3]. Ionic and nanoparticle metals have been proposed, including silver, copper, excess iron (II, III) and gallium. Unfortunately, many of these studies were conducted by material scientists and engineers who had little understanding of the evolutionary dynamics of populations exposed to toxic materials [4, 5]. These studies claimed the fact that metals impacted so many different aspects of bacterial physiology, that they represented steep obstacles to the evolution of resistance [4].

Recently several studies have examined the impact of the iron analog gallium upon the growth of pathogenic bacteria [6]. The mechanism targeted by this approach was to use gallium (Ga^3+^) to saturate bacterial siderophores that normally take up ferric iron (Fe^3+^). Studies on the impact of gallium suggest that it may impede growth without killing cells (bacteriostatic) or eventually kill cells via prolonged iron starvation (bactericidal) [6]. Gallium’s (Ga^3+^) capacity to quench bacterial siderophores is due to this element’s similarity with ionic radius and binding properties of iron (III, Fe^3+^) [6]. Gallium is a group IIIA metal with atomic number 31 and shares certain properties with Fe^3+^, where the octahedral ionic radius of Ga^3+^ is 0.620 Å compared with 0.645 Å for Fe^3+^ and the tetrahedral ionic radius is 0.47 Å for Ga^3+^ compared with 0.49 Å for Fe^3+^ [7].

It is known that iron (as well as other micronutrient metals Mg, Mn, S and Zn) play key roles in the capacity for pathogenic bacteria to maintain their virulence. These metals often serve as enzymatic co-factors. Therefore, their intracellular concentration must be maintained and tightly regulated to maintain cell viability [8]. Iron is one of the most important micronutrients as it fulfills many biological roles [9]. Iron-containing proteins (heme-proteins, iron-sulfur cluster proteins and di-iron and mononuclear enzymes) play roles in nitrogen fixation and metabolism and serve as electron carriers for respiration [10]. Despite its critical role in bacterial metabolism, acquiring iron is one of the greatest challenges for bacterial growth [8, 11] and iron deficiency is one of the most common nutritional stresses [12]. Despite iron being an essential metal, it can also be extremely toxic under aerobic conditions [13].

In aerobic environments, iron predominantly occurs as ferric iron (Fe^3+^), however, Fe(OH)_3_ is poorly soluble in aqueous solution (as low as 10^−18^ M at pH 7.0). Under anaerobic conditions, the equilibrium shifts to ferrous iron (Fe^2+^) that is more readily bioavailable to microorganisms and availability is a key to pathogenesis for a variety of microbes, thus many innate immunity mechanisms utilize iron sequestration (e.g. serum albumin, calprotectin [14, 15]). As iron is essential, microbes have evolved means to take it up from their extracellular surroundings; for example, siderophores. The siderophore, enterobactin, in *Escherichia coli* is synthesized via genes such as *entC* and *fep* genes (in strain K-12: A, B, C, D, E and G). These siderophores are tightly controlled by the global iron homeostasis regulator, Fur [8]. Under aerobic conditions, excess iron has been shown to induce oxidative damage, whereas both Fe^2+^ and Fe^3+^ can interact with hydrogen peroxide and superoxides, respectively, which generate highly reactive hydroxyl radicals leading to cell damage and eventual cell death [13]. This is in addition to a variety of other effects summarized in Table 1. When oxygen free radicals form in their cells, bacteria have genes to defend themselves from the accrued stressors. This is aided by many proteins including OxyR that responds to the presence of hydrogen peroxide, SoxS and SoxR that respond to redox active compounds and RpoS that responds to general oxidative stress [17–19]. It is therefore important that intracellular iron be regulated to prevent toxicity by this essential metal [20, 21].

**Table 1.** Mechanisms of excess iron and gallium toxicity

Mechanism	Fe	Ga
Reactive oxygen species	+	+
Disruption of transcription/translation	+	−
Damage to cell wall/membrane	+	+
Interfering with respiration	+	+
Release of cellular components	+	+
Binding to thiol groups	?	?
Change in zinc homeostasis	−	+
Disruption of iron metabolism	−	+

Mechanisms of cellular damage are listed resulting from excess iron toxicity. As these systems are common to virtually all bacteria, there is a strong potential that resistance mechanisms might be conserved across wide varieties of taxa. +, established; ?, unknown. Sources: [7, 11, 16].

We have recently shown that *E. coli* can evolve resistance to excess iron (II and III) [22, M. D. Thomas, J. L. Graves, A. J. Ewunkem *et al.* in preparation]. Furthermore, an unexpected result of excess iron resistance in these strains was resistance to gallium which is an iron analog possessing the same nuclear radius as iron (Fe^3+^). For this reason, gallium has been shown to exchange for iron in siderophores such as in *Pseudomonas aeruginosa* where the siderophore pyochelin transports gallium into the cell [23], it also represses *pvdS*, a transcriptional regulator of the siderophore pyoverdine [6]. Once inside the cell gallium cannot be used for essential iron catalyzed reactions and is therefore inhibits growth (bacteriostatic) due to mismetallation. In addition, gallium has been shown to perturb the regulation of iron acquisition pathways and can eventually cause cell death [6].

Until recently, experiments showing the evolution of gallium resistance were limited to *Pseudomonas* [24–26]. However, a recent study utilized the Keio collection which consists of 3985 single, non-essential gene knockouts in *E. coli* BW25113 and examined the effect on gallium resistance [27, 28]. Each mutant was scored as to whether it increased or decreased gallium sensitivity on a scale of −0.6 to +0.6 normalized to colony size. The mutants were classified by their impact on cellular metabolism (transcription, translation, DNA metabolism, RNA metabolism, protein metabolism, protein folding and secretion, cell exterior functions, biosynthetic functions, degradation, energy, cellular processes, response to stimulus and other pathways). Of the 3945 mutations studied, they found a relatively equal distribution of mutations that raised or lowered gallium sensitivity (1761 raised and 1878 lowered). However, the vast majority of these effects on colony size were minor. These sorts of studies are limited in their capacity to predict how bacteria might actually evolve resistance to gallium or any stressing material, as the gene knockouts were of non-essential functions. Furthermore, knocking out a gene may produce unanticipated pleiotropic effects on fitness that are hard to interpret in any given environment.

Therefore, in this study we utilize experimental evolution on *E. coli* K-12 MG1655 to ask whether and how does *E. coli* evolve resistance to excess gallium. In addition, since we have already shown that this strain can evolve excess iron (II, III) resistance, we wanted to know whether gallium resistant *E. coli* would also have a correlated response that confers iron (II, III) resistance. Furthermore, we asked whether gallium resistance confers resistance to metals that may not have similar bacteriostatic/bactericidal effects to gallium such as silver (Table 1). We also examined the genomic changes, that allow *E. coli* to survive in environments containing excess gallium and compared those with the knockout results of Gugala *et al.* [28]. Finally, we modeled the structural changes in one of the proteins altered by selection to propose a mechanism by which this mutation conferred gallium resistance.

## METHODOLOGY

### Evolution experiment

Experimental evolution is defined as the study of populations over defined and reproducible conditions over multiple generations under laboratory or natural conditions [29]. It has been used to study adaptation in a variety of organisms and conditions, including the study of antimicrobial resistance via antibiotics (ampicillin, beta lactams, chloramphenicol and others), as well as metals [30–34]. Other methods have been used, such as step-wise selection to isolate metal resistant mutants in *E. coli* [35]. However, the underlying principle of artificial selection is the same as that employed in experimental evolution. However, our method allows the study of adaptation to increase antimicrobial metal concentration over time, which does not result from the step-wise selection protocol.

*Escherichia coli* K-12 MG1655 (ATCC #47076) was chosen for this study due to the paucity of known metal resistant loci in this bacterium. This is also the strain we have used in our previous studies evaluating the evolution of silver and iron resistance [21, 22, 33, 34]. Its chromosome has 4 641 652 nucleotides (GenBank: NC_000913.3) and contains no plasmids. We have sequenced this strain and polymorphisms not present in the published reference genome are given in in previous studies [33, 34].

In our experiments, *E. coli* was cultured in Davis Minimal Broth (DMB; Difco, Sparks, MD) with 1 g of dextrose per liter (Fisher Scientific, Fair Lawn, NJ) carbon source. The medium is enriched with thiamine hydrochloride with 10 µl in the final volume of 10 ml, for a concentration of 0.3 µM. This is kept in 50-ml Erlenmeyer flasks and placed in a shaking incubator at 115 rpm at 37°C. Each replicate was founded from a unique colony on an agar plate isolated via serial dilution from the ancestral stock culture. These samples were placed in separate flasks and allowed to grow for 24 hours before being transferred to fresh media. Five replicates of the controls (C_1_–C_5_) which are grown in standard DMB medium without the addition of gallium and five replicates of the Ga(NO_3_)_3_ selected (Ga^3+^_1–Ga^3+^_5) were founded and propagated by subculturing 0.1 ml into 9.9 ml of fresh sterile DMB daily. The population density increased from ∼10^7^ to ∼10^9^ cells per ml over the course of each daily transfer cycle. Replication of selection treatments is important because it allows for the observation of parallelism, that is, adaptations that are general responses to the selection regime.

A minimum inhibitory concentration (MIC) assay utilizing the ancestral *E. coli* K-12 MG1655 strain allowed the determination of the sublethal concentration of each gallium nitrate to be utilized for selection purposes. This was determined to be 100 mg/l of Ga(NO_3_)_3_ during 10 days of culture. The gallium selected and control populations were frozen at −80°C for future analysis. To determine the potential impact of the nitrate ion (${\text{NO}}_{3}^{-}$) on the growth of the controls and gallium nitrate selected populations, a MIC assay was conducted on both populations in sodium nitrate (NaNO_3_). The reduction in 24-hour growth as measured by optical density at 625 nm from 6.25 to 500 mg/l was 0.012 for the controls and 0.014 for the gallium-selected populations. From these results, we determined that resistance to the nitrate ion would not be a significant factor in the experiment.

### Phenotypic assays: 24-hour growth

Measurements of 24-hour growth in excess metal (Ga^3+^, Fe^2+^, Fe^3+^ and Ag^+^) and traditional antibiotics for the ancestral, gallium-selected and control populations were determined at 10 days of evolution. These values were compared with the five samples of the *E. coli* K-12 MG1655 ancestor which was grown over night in standard DMB medium. The range used was 0–1000 mg/l for the Ga^3+^; 0–1750 mg/l for the Fe^2+^ and Fe^3+^ assays, 0–100 mg/l for Ag^+^ and finally 0–500 mg/l for the antibiotics (chloramphenicol, rifampicin, sulfanilamide and tetracycline). Previous studies of *E. coli K-12* MG1655 have shown that this strain has no resistance to these antibiotics [36]. Statistical analysis of the effect of selection regime and concentration (and their interaction) for all 24-hour growth data was performed via General Linear Model utilizing SPSS version 23 (SPSS Inc, Armonk, NY, USA). All graphs in this paper were made via SigmaPlot version 14. Finally, the phenotypic data from these studies will be submitted into DRYAD (https://datadryad.org/) upon acceptance of this manuscript for publication.

### Genomic analysis

DNA was extracted from each population after 10 days of culture using the EZNA Bacterial DNA extraction kit (Omega Bio-tek®) as per manufacturer instructions. DNA concentrations were normalized using the QuantiFluor® dsDNA system. Genomic libraries were prepared using the Illumina Nextera XT kit and samples were sequenced using the Illumina MiSeq sequencing platform. The depth of coverage of the sequencing runs ranged from ∼20× to ∼80×, with most exceeding 40× coverage. The SRA accession number for sequencing data from this study are PRJNA521749 (Ga^3+^-resistant and controls).

Sequence alignment and variant calling from the samples was achieved by use of the *breseq* 0.30.0 pipeline [37]. The breseq pipeline uses three types of evidence to predict mutations, read alignments, missing coverage and new junctions, and any reads that indicate a difference between the sample and the reference genome that cannot be resolved to describe precise genetic changes are listed as ‘unassigned’ [37]. These unassigned reads are not described or interpreted here.

The position at which the FecA mutations occurred were then identified in the published structure of FecA in complex with iron-citrate (PDB1PO3) [38] and visualized using PYMOL [39]. This was done in order to predict the functional consequences of the identified mutations.

## RESULTS

### Phenotypic changes in presence of metals

**Figure 1.**
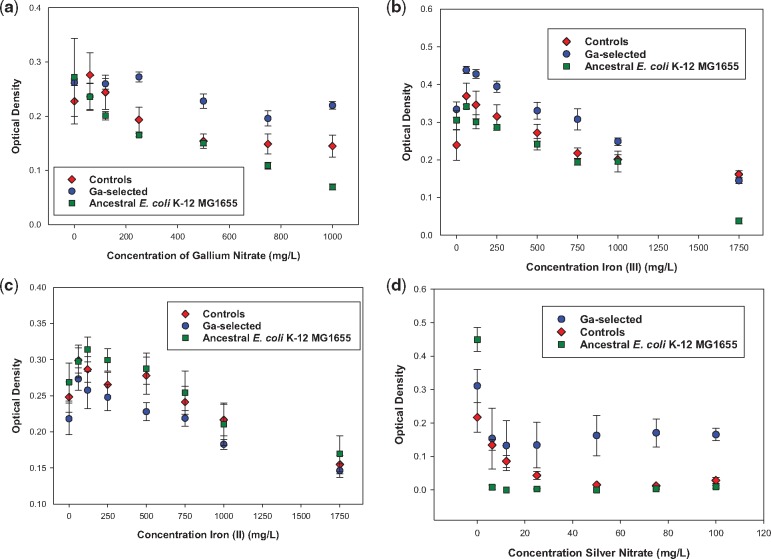
(**a**) The mean and SE of 24-hour growth for populations in increasing concentration of gallium (Ga3+) to 1000 mg/l after 10 days of evolution. Ga3+ selected were significantly > controls = ancestors. (**b**) The mean and SE of 24-hour growth for populations in increasing concentration of iron III (Fe3+) to 1750 mg/l after 10 days of evolution. Ga^3+^ selected were significantly > controls = ancestors, the ancestors had virtually no ability to grow in iron (III) at the highest concentration measured. (**c**) The mean and SE of 24-hour growth for populations in increasing concentration of iron II (Fe2+) to 1750 mg/l after 10 days of evolution. The controls = ancestors were significantly > than the Ga3+-selected populations. (**d**) The mean and SE of 24-hour growth for populations in increasing concentration of silver (Ag+) to 100 mg/l after 10 days of evolution. The Ga3+-selected were significantly > controls > ancestors

Figure 1a–d shows 24-hour growth across increasing concentrations of gallium, iron (II), iron (III) and silver nitrate. The Ga^3+^-selected populations showed superior growth relative to the controls and ancestral strain across concentration for gallium, iron (III) and silver nitrate. The controls and ancestral populations showed superior growth relative to the Ga^3+^-selected populations in iron (II). For all substances tested, the Ga^3+^-selected, controls and ancestral population decreased their 24-hour growth with increased concentration of the metal tested. There were two significant interactions between the population and concentration variables, in gallium and iron (II) resistance comparisons between the Ga^3+^-selected and ancestral population. The F-statistics and *P*-values for all comparisons are given in Table 2.

**Table 2.** F statistics and *P*-values for phenotypic tests

Substance	Range tested	Population	Concentration	Interaction
Gallium resistant > Controls				
Gallium Ga(NO_3_)_3_	200–1000 mg/l	F = 11.42, *P* = 0.0001	F = 5.00, *P* = 0.001	F = 0.81, *P* = 0.99, NS
Iron (III) Fe_2_(SO4)_3_	6.2–1750 mg/l	F = 30.40, *P* = 0.0001	F =27.41, *P* = 0.0001	F = 1.22, *P* = 0.30, NS
Silver nitrate AgNO_3_	6.2–100 mg/l	F = 16.00, *P* = 0.0001	F = 3.83, *P* = 0.003	F= 0.06, *P* = 0.99, NS
Gallium resistant > Ancestor				
Gallium Ga(NO_3_)_3_	200–1000 mg/l	F= 110.64, *P* = 0.0001	F = 18.09, *P* = 0.0001	F = 7.18, *P* = 0.0001
Iron (III) Fe_2_(SO4)_3_	6.2–1750 mg/l	F= 139.00, *P* = 0.0001	F= 84.15, *P* = 0.0001	F = 1.13, *P* = 0.357, NS
Silver nitrate AgNO_3_	6.2–100 mg/l	F = 33.10, *P* = 0.0001	F = 0.08, *P* = 0.995, NS	F = 13.61, *P* = 0.001
Rifampicin	6.2–25 mg/l	F = 37.0, *P* = 0.0001	F = 7.7, *P* = 0.0001	F = 1.1, *P* = 0.356
Sulfanilamide	100–250 mg/l	F = 20.8, *P* = 0.0001	F = 0.877, *P* = 0.431	F = 0.238, *P* = 0.790
Gallium resistant = controls				
Chloramphenicol	6.2–250 mg/l	F = 1.81, *P* = 0.182	F = 19.68, *P* = 0.0001	F = 0.35, *P* = 0.92, NS
Sulfanilamide	6.2–250 mg/l	F = 0.287, *P* = 0.532	F =0.532, *P* = 0.807	F = 0.829, *P* = 0.56, NS
Tetracycline	6.2–250 mg/l	F = 0.135, *P* = 0.885	F = 0.85, *P* = 0.526	F = 155, *P* = 0.171, NS
Gallium resistant = ancestor				
Chloramphenicol	6.2–250 mg/l	F = 0.355, *P* = 0.554	F = 2.88, *P* = 0.01	F = 0.170, *P* = 0.99
Sulfanilamide	6.2–75 mg/l	F = 0.01, *P* = 0.907	F = 0.30, *P* = 0.870	F = 1.44, *P* = 0.23
Tetracycline	6.2–250 mg/l	F = 0.504, *P* = 0.481	F =1.20, *P* = 0.27	F = 1.27, *P* = 0.28
Rifampicin	50–250 mg/l	F = 0.07, *P* = 0.79	F = 1.3, *P* = 0.27	F = 3.0, *P* = 0.03
Controls > gallium resistant				
Iron (II) Fe_2_SO_4_	6.2–1750 mg/l	F = 8.98, *P* = 0.004	F =11.44, *P* = 0.0001	F = 0.22, *P* = 0.97, NS
Rifampicin	6.2–250 mg/l	F = 33.51, *P* = 0.0001	F =7.75, *P* = 0.0001	F = 0.68, 0.60, NS
Controls > Ancestor				
Gallium	200–1000 mg/l	F = 9.34, *P* = 0.003	F = 13.55, *P* =0.0001	F = 0.568, *P* = 0.724, NS
Iron (III) Fe_2_(SO4)_3_	6–1750 mg/l	F = 13.62, *P* = 0.001	F = 36.16, *P* = 0.0001	F = 1.72, *P* = 0.133, NS
Silver nitrate	6–100 mg/l	F = 82.83, *P* = 0.0001	F =13.92, *P* = 0.0001	F = 13.6, *P* = 0.001
Rifampicin	6–250 mg/l	F = 309.5, *P* = 0.0001	F = 7.7, *P* = 0.0001	F = 2.6, *P* = 0.019
Sulfanilamide	100–250 mg/l	F = 19.5, *P* = 0.0001	F = 0.07, *P* = 0.93	F = 0.63, *P* = 0.54
Controls = Ancestor				
Iron (II) Fe_2_SO_4_	6–1, 750 mg/l	F = 0.05, *P* = 0.813,	F = 30.13, *P* = 0.0001	F = 3.72, *P* = 0.003
Chloramphenicol	6–250 mg/l	F = 2.83, *P* = 0.09	F = 3.28, *P* = 0.005	F= 0.559, *P* = 0.78
Sulfanilamide	6–75 mg/l	F = 0.87, *P* = 0.355	F = 3.51, *P* = 0.015	F = 0.436, *P* = 0.781
Tetracycline	6–250 mg/l	F = 0.004, *P* = 0.952	F = 1.19, *P* = 0.323	F = 0.886, *P* = 0.525
Ancestor > gallium resistant				
Iron (II) Fe_2_SO_4_	6.2–1750 mg/l	F = 36.22, *P* = 0.0001	F =30.13, *P* = 0.0001	F = 3.72, *P* = 0.003

**Figure 2.**
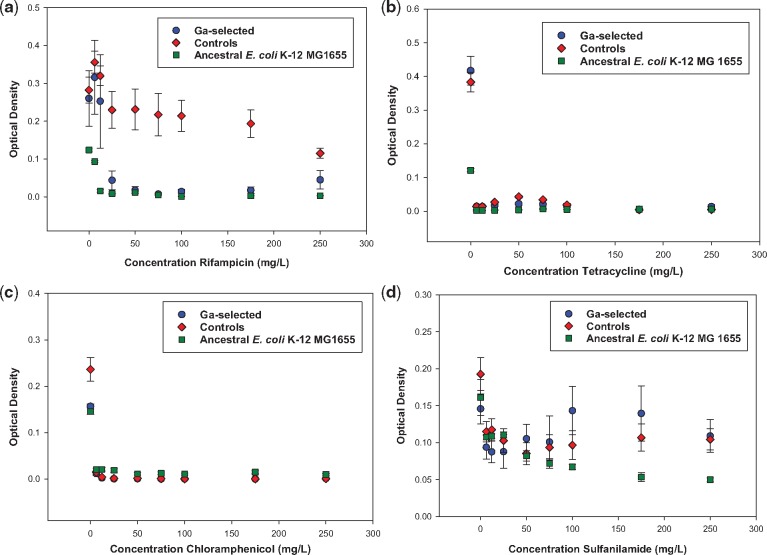
(**a**) The mean and SE of 24-hour growth for populations in increasing concentration of rifampicin to 250 mg/l after 10 days of evolution. The controls from 25 to 250 mg/l were significantly > then the Ga3+-selected populations. The Ga3+-selected > ancestral from 6 to 25 mg/l and were equivalent from 50 to 250 mg/l. (**b**) The mean and SE of 24-hour growth for populations in increasing concentration of tetracycline to 250 mg/l after 10 days of evolution. The controls, Ga3+-selected and ancestral populations effectively showed no growth from 6 to 250 mg/l. (**c**) The mean and SE of 24-hour growth for populations in increasing concentration of chloramphenicol to 250 mg/l after 10 days of evolution. There was effectively no growth and no difference between the Ga3+-selected populations, controls and the ancestors from 6 to 250 mg/l. (**d**) The mean and SE of 24-hour growth for populations in increasing concentration of sulfanilamide to 250 mg/l after 10 days of evolution. There was no difference between the Ga3+-selected, controls and ancestral populations from 6 to 75 mg/l; however, the Ga3+-selected = controls and both were significantly > than the ancestral populations from 100 to 250 mg/l

The 24-hour growth of the Ga^3+^-selected, control and ancestral populations from 0 to 250 mg/l in rifampicin, tetracycline, chloramphenicol and sulfanilamide are shown in Figure 2a–d. The control populations showed superior growth relative to the Ga^3+^-selected and ancestral population in increasing concentrations of rifampicin. There was no significant difference between the selected, control and ancestral populations in chloramphenicol and tetracycline (growth ceased at 6.2 mg/l). There was no difference between Ga^3+^-selected, control and ancestral populations from 6 to 75 mg/l of sulfanilamide. However, from 100 to 250 mg/l, both the Ga^3+^-selected and control populations showed equivalent growth which was statistically significantly greater than that of the ancestral population.

### Genomic results

Table 3 shows selective sweeps in the Ga^3+^-resistant populations at day 10. There were eight hard selective sweeps (0.000 ancestor–1.000 in the Ga^3+^-resistant populations); one hard sweep found in (Ga^3+^_1, Ga^3+^_2 and Ga^3+^_5), Ga^3+^_3 displayed two hard sweeps and Ga^3+^_4 showed four hard sweeps. Of the hard sweeps, all populations showed a mutation in *fecA* (UniProtKB-P77279). Ga^3+^_1, Ga^3+^_2, Ga^3+^_3 and Ga^3+^_5 showed the same mutation, and Ga^3+^_4 had a different mutation in this gene. Ga^3+^_3 showed a hard selective sweep in *insl1* (UniProtKB-P0CF91), Ga^3+^_4 in *yedN* (UniProtKB-A0A023BD4). There was one hard sweep in an intergenic region (*qmcA* ←/→ *fetA*). The *qmcA* (UniProtKB-P0AA53) gene encodes a prohibitin homology domain-anchored (PHB) domain-anchored putative protease and *fetA* (UniProtKB-P77279) encodes an iron exporter, ATP binding subunit, ABC transporter FETAB subunit; peroxidase resistance protein. There were seven significant polymorphisms (0.351–0.39) distributed across the five populations. Ga^3+^_1 and Ga^3+^_3 show an identical mutation in *fecE* (dicitrate transport ATP binding protein, UniProtKB-P15031). Ga^3+^_4 shows a mutation in *fecB* (binds citrate-dependent Fe^3+^; part of the binding protein-dependent transport system for uptake of citrate-dependent Fe^3+^, UniProtKB-P15028). Ga^3+^_3 displays a mutation in *ydfE* (Qin prophage, pseudogene; phage or prophage related, UniProtKB-Q7138). The remaining significant polymorphisms are intergenic, Ga3+_1 (*fepD* ←/→ *entS*; *fepD* UniProtKB-23876 is part of the binding protein-dependent transport system for ferric enterobactin. Probably responsible for the translocation of the substrate across the membrane; *entS* UniProtKB-24077 has enterobactin transmembrane transporter activity, also involved in cellular response to DNA damage stimulus, and response to antibiotic). Ga^3+^_3 has an intergenic mutation [*yfgF* ←/→ *yfgG*; *yfgF* UniProtKB-P77172 is a phosphodiesterase (PDE) that catalyzes the hydrolysis of cyclic-di-GMP (c-di-GMP) to 5′-pGpG; yfgG UniProtKB-P64545 is an uncharacterized protein]. Finally, six low-level polymorphisms were observed. Ga^3+^_1 displayed a mutation in *prlF* UniProtKB-15373, antitoxin component of a type II toxin-antitoxin (TA) system, Ga^3+^_2 had a mutation in *nhaB* UniProtKB-P0AFA7, a Na^+^/H^+^ antiporter that extrudes sodium in exchange for external protons and Ga^3+^_5 had one in *rpoS* UniProtKB-P13445, RNA polymerase sigma factor S. The remaining low-level polymorphisms were intergenic with Ga^3+^_1 showing *ypjC* ←/← *ileY*; *ypjC* UniProtKB-76613 predicted protein and *ileY* tRNA isoleucine; *agaI* →/→ *yraH*, *agaI* UniProtKB-P42912, putative deaminase, galactosamine-6 phosphate isomerase; *yraH* UniProtKB-P42913, putative fimbrial-like adhesin protein; *dcuB ←/← dcuR*, *dcuB*, UniProtKB-P0ABN9, anaerobic C4-dicarboxylate transporter DcuB and DcuR are members of the two-component regulatory system DcuB/DcuR, involved in the C4-dicarboxylate-stimulated regulation of the genes encoding the anaerobic fumarate respiratory system. Table 4 and 5 report the annotation and functional description of these genes. Of the selective sweeps in these populations, six were non-synonymous single nucleotide polymorphisms, seven were intergenic, two were insertions in coding regions and two were insertions in pseudogenes.

**Table 3.** Selective Sweeps in Ga^3+^-resistant populations at day 10

Gene	Position	Mutation	Ga1	Ga2	Ga3	Ga4	Ga5
qmcA ← / → fetA	515, 859	C→G	0.000	0.000	0.000	1.000	0.000
fepD ← / → entS	622, 244	IS5 (–) +4 bp	0.351	0.000	0.639	0.000	0.000
nhaB ←	1, 234, 632	A→G	0.000	0.190	0.000	0.000	0.000
ydfE →	1, 650, 461	C→A	0.000	0.000	0.452	0.000	0.000
yedN ←	2, 011, 665	G→T	0.000	0.000	0.000	1.000	0.000
yfgF ← / → yfgG	2, 629, 042	IS5 (+) +4 bp	0.000	0.000	0.500	0.000	0.000
ypjC ← / ← ileY	2, 785, 563	G→A	0.193	0.000	0.000	0.000	0.000
rpoS ←	2, 866, 695	IS1 (+) +9 bp	0.000	0.000	0.000	0.000	0.273
prlF →	3, 277, 273	(TTCAACA)2→3	0.184	0.000	0.000	0.000	0.000
agaI → / → yraH	3, 287, 319	C→T	0.161	0.000	0.000	0.000	0.000
dcuB ← / ← dcuR	4, 349, 066	T→A	0.147	0.000	0.000	0.000	0.000
insI1 ←	4, 507, 739	G→T	0.000	0.000	1.000	0.000	0.000
fecE ←	4, 510, 928	A→T	0.514	0.000	0.574	0.000	0.000
fecB ←	4, 514, 119	C→T	0.000	0.000	0.000	0.479	1.000
fecA ←	4, 515, 480	C→T	1.000	1.000	1.000	0.000	1.000
fecA ←	4, 516, 171	G→C	0.000	0.000	0.000	1.000	0.000

Color coding: yellow, fixation; green, major variant; blue, minor variant.

**Table 4.** Annotation of genes in Table 2a

Gene	Annotation
qmcA ← / → fetA	intergenic (-86/-60)
fepD ← / → entS	intergenic (-55/-53)
nhaB ←	L29S (TTA→TCA)
ydfE →	pseudogene (384/765 nt)
yedN ←	pseudogene (376/678 nt)
yfgF ← / → yfgG	intergenic (-104/-245)
ypjC ← / ← ileY	intergenic (-552/+199)
rpoS ←	coding (849-857/993 nt)
prlF →	coding (272/336 nt)
agaI → / → yraH	intergenic (+294/-107)
dcuB ← / ← dcuR	intergenic (-322/+249)
insI1 ←	D293E (GAC→GAA)
fecE ←	L177Q (CTG→CAG)
fecB ←	D64N (GAT→AAT)
fecA ←	G400S (GGC→AGC)
fecA ←	N169K (AAC→AAG)

Color coding: blue, missense mutation; red, nucleotides changed.

**Table 5.** Description of genes in Table 2a

Gene	Description
qmcA ← / → fetA	PHB domain membrane-anchored putative protease/iron exporter^a^
fepD ← / → entS	Iron-enterobactin transporter subunit/enterobactin exporter^b^
nhaB ←	Sodium: proton antiporter
ydfE →	Qin prophage; pseudogene; Phage or Prophage Related
yedN ←	Pseudogene, IpaH/YopM family
yfgF ← / → yfgG	Cyclic-di-GMP PDE^c^
ypjC ← / ← ileY	Pseudogene/tRNA-Ile
rpoS ←	RNA polymerase, sigma S (sigma 38) factor
prlF →	Antitoxin of the SohA(PrlF)-YhaV TA system
agaI → / → yraH	Galactosamine-6-phosphate isomerase^d^
dcuB ← / ← dcuR	C4-dicarboxylate transporter, anaerobic; DcuS co-sensor^e^
insI1 ←	IS30 transposase
fecE ←	Iron-dicitrate transporter subunit E
fecB ←	Iron-dicitrate transporter subunit B
fecA ←	Ferric citrate outer membrane transporter

Genes that are directly related to iron metabolism are highlighted in red.

a ATP-binding subunit, ABC transporter FetAB subunit; peroxide resistance protein.

b Iron-regulated.

c Anaerobic/uncharacterized protein.

d Putative fimbrial-like adhesin protein.

e Response regulator in two-component regulatory system with DcuS.

The most notable mutations observed in the control populations were in an intergenic mutation occurring between *arsC* →/→ *yhiS* in the C_4_ population; and a series of mutations in *rpoB* → in C_1_–C_3_, at position 4 183 378 ranging in frequency from 0.655 to 0.320; in C_4_ at position 4 182 820 in a frequency of 0.338 and in C_4_ at position 4 183 379 in frequency of 0.607. The *arsC* (UniProtKB-POAB96) gene is involved in reducing arsenate to arsenite allowing for efflux of this toxic material from the cell and *yhiS* is an uncharacterized protein. RNA polymerase subunit B (*rpoB*) is a DNA-dependent RNA polymerase that catalyzes the transcription of DNA into RNA using the four ribonucleoside triphosphates as substrates; and resistance to the antibiotics salinamide A, salinamide B, rifampicin, streptolydigin, CBR703, myxopyronin and lipiarmycin can result from mutations in this protein (UniProtKB-POA8V2).

All polymorphisms classified by breseq in the Ga^3+^-selected populations and the controls are found in Supplementary Tables 1a–c and 2a–c. None of the suspected mutations associated with Ga^3+^ resistance were found in the controls, or have ever been observed in the control populations of our previous studies [21, 22, 33, 34].

## DISCUSSION

These studies have clearly shown that *E. coli* K-12 MG1655 can rapidly evolve resistance to gallium. We predicted that this would be true based upon our previous work that demonstrated that this strain could evolve resistance to excess iron (II, III) and that in each case, increased resistance to gallium resulted [21, 22]. Our result is in direct opposition to previously published claims concerning gallium quenching as an evolutionarily stable antimicrobial treatment [24]. That study examined experimental evolution of gallium resistance and found no evidence of anti-gallium resistance over the course of 12 days for *P. aeruginosa*. On the face of it that result is counter intuitive, given that a 2013 study did find evidence of anti-gallium mutations in *P. aeruginosa* [26] and a recent 2018 study corroborated the 2013 result [25]. Thus, it seems clear that Gram-negative bacteria do have the capacity to evolve resistance to gallium quenching.

In addition to gallium resistance, correlated resistance to excess iron (III, Fe^3+^) and ionic silver (Ag^+^) was observed in the Ga^3+^-resistant populations. The latter result can be questioned, in that the genomic analysis showed no selection in genes that have previously been associated with silver resistance. Although selection for iron (II) and iron (III) resistance in our previous studies did confer a minor increase in silver resistance without evidence of selection in the genes *cusS*, *ompR*, that played a major role in conferring resistance [21, 22, 33, 34]. Therefore, it is possible that gallium selection is conferring a minor increase in silver resistance by mechanisms, we do not as of yet understand. Finally, our Fe^2+^- and Fe^3+^-resistant populations showed cross resistance to iron (II) and iron (III). The Ga^3+^-resistant populations are resistant to iron (III), but not to iron (II).

The Ga^3+^-selected populations also showed inferior 24-hour growth relative to the controls and the ancestors in the traditional antibiotic rifampicin. This can be linked to the selective sweeps in *rpo*B that were found in the control populations, but not in the Ga^3+^-resistant populations or ancestors. Mutations in *rpoB* are commonly observed in *E. coli* experimental evolution, including adaptation to media-containing minimal amounts of carbon source (glucose/lactose) and these are associated with increased resistance to rifampicin [40–42]. It seems that pleiotropic effects of such mutations may be a crucial means by which the evolution of antibiotic resistance proceeds in the absence of antibiotics [43, 44].

There was no significant difference between the Ga^3+^-resistant, control and ancestral populations in chloramphenicol and tetracycline. The Ga^3+^-resistant, control and ancestral populations showed equivalent growth at lower concentrations of sulfanilamide (6–75 mg/l). However, both the Ga^3+^-resistant and control populations performed better at higher concentrations (100–250 mg/l) of sulfanilamide compared with the ancestor. As neither of these populations were exposed to sulfanilamide, it is likely that this improvement in growth is due to some other aspect of the environment shared by both populations (such as minimum sugar content). ATCC cultures this strain using Luria broth which has a higher sugar content than DMB (https://www.atcc.org/products/all/47076.aspx#culturemethod).

The lack of correlated antibiotic resistance in the Ga^3+^-resistant populations is significant in that our iron-selected populations (Fe^2+^ and Fe^3+^) did show correlated resistance to antibiotics [21, 22]. The difference between these strains in correlated responses to selection is best explained by the differences in the genomic foundations of their respective resistances. All the Ga^3+^-resistant populations showed a mutation in the *fecA* gene (ferric citrate outer membrane transporter, the G400S mutation was observed in all populations except Ga^3+^_4 which displayed N169K). This gene encodes the protein FecA is responsible for Fe^3+^ transport in aerobic conditions [11]. We propose that these mutations reduce the function of FecA, thereby limiting Ga^3+^ import in these strains. For example, we have shown the FecA mutations (A559T and G243C) observed in our iron (II)-resistant populations express 128-fold less *fecA* than the controls in absence of iron indicating a likely preemptive mechanism of defense [22].

Several of the other genomic variants showing strong selection involved in conferring gallium resistance are in genes and intergenic regions associated with iron metabolism (*fecE; fecB; qmcA/fetA; fepD/entS*). The remaining polymorphisms showing evidence of selection have no clear relationship to iron metabolism, thus some could be responses to excess NO_3__−_ in the medium, or they could have yet undiscovered relationships to gallium resistance. It is also important to note the difference in our genomics results (via experimental evolution) and those obtained via knock out mutations in *E. coli* [9] in that none of our genes resulting from selection for gallium resistance were found in the previous study. This results from the fact that the Keio strains were produced by knockout of non-essential genes in *E. coli* K-12 [27] and our selective sweeps occurred in genes essential to iron metabolism in *E. coli* K-12 (e.g. *fecA*, *fecE* and *fecB*). In addition, the knock out method cannot identify intergenic regions that contribute to gallium adaptation, as no intergenic regions are knocked out in these strains. Thus, at best, knock out methods can identify genes that could possibly play a role in adaptation, but this technique is not necessarily going to tell you which genes will play a role in adaptation.

Our studies of experimental evolution of iron resistance showed that co-selection of metal and antibiotic resistance, and this can occur from linkage as well as pleiotropy. In the case of metals and antibiotics, pleiotropy is well known. For example, resistance mechanisms to metals such as reduction in cell wall permeability, substance alteration, efflux, alteration of cellular targets and sequestration are wide spread, and these traits also produce resistance to traditional antibiotics [45, 46].

**Figure 3.**
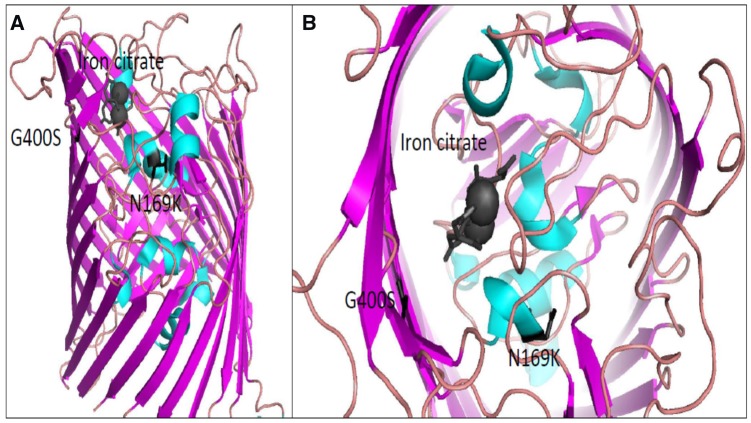
(a) Depicts a full view of the FecA protein, iron-citrate is bound on the periplasmic side and colored dark grey. Both the G400 and N169 are colored black as their mutation to G400S and N169K were identified to play a role in conferring gallium resistance. (b) Close up view of the FecA protein from the top of the beta-barrel shows that G400 is directly adjacent to iron-citrate. N169 sits on a helix which is in direct contact also with iron-citrate. Figures were generated using PDB1P03 in PyMOL.

However, our Ga^3+^-resistant lines, do not display pleiotropy between gallium and antibiotic resistance whereas our iron (II, III) populations showed positive pleiotropy for five antibiotics: ampicillin, chloramphenicol, rifampicin, sulfanilamide and tetracycline [33, 34]. Genomic analysis illustrated that four of the five Fe^2+^-selected replicates showed at least one or more of these populations identified hard sweeps in genes such as *murC, cueR, tolC, yeaG and ptsP.* Several of these genes have been shown to be associated with antibiotic resistance, such as in the case of the *murC* which plays a role in cell wall biosynthesis as well as repairing oxidative damage [47]. However, the gallium-resistant populations do not display any selective sweeps in genes with an established relationship to antibiotic resistance, whereas the controls did display mutations in *rpoB*, that are consistently associated with antibiotic resistance [40–42]. We believe that the most functionally relevant mutations observed in this study is in FecA. As previously stated, FecA, through the siderophore citrate, is an iron (III) transporter under aerobic conditions. FecA is a transmembrane β-barrel and its structures has previously been solved both in its apo form and in complex with iron citration [48]. We used the complexed structure (PDB1PO3) as a guide to predict functional outcomes of the observed mutations. Both residues identified in this study (G400S and N169K) were found directly in the ligand binding pocket (Figure 3). The G400 amino acid sits directly adjacent to iron-citrate and the introduction of a polar group through mutation to a serine could easily hinder binding of the siderophore to the pocket. The second residue N169 sits on the backside of a helix which is also in contact with the iron citrate. The mutation to a large and positively charged amino acid such as lysine could potentially displace this helix into the ligand-binding pocket, also occluding entry of the siderophore. These mechanisms remain to be evaluated.

In summary, we have found that *E. coli* K-12 MG1655 can rapidly evolve resistance to gallium in opposition to claims of the evolutionary stability of gallium quenching. This trait also conferred resistance to ferric iron (iron III) and ionic silver, but no correlated responses to antibiotics were observed. The genomic results indicated that gallium resistance results from selection on mutations associated with iron metabolism. This is not surprising as gallium is an iron analog. We propose that these mutations are likely to be associated with the down regulation of genes involved in iron acquisition; as we observed in our iron (II)- and iron (III)-resistant populations [33, 34].

As in the evolution of silver, copper and iron resistance, a small number of genomic changes were required to produce the gallium-resistant phenotypes. All replicates showed at least one hard selective sweep (*fecA*). The parallelism of the mutations in *fecA* is a strong evidence that this gene played a major role in conferring gallium resistance. The Ga^3+^_4 replicate showed three additional hard sweeps, while the other populations had associated polymorphisms associated with iron metabolism not observed in the ancestor or control populations. We also suspect that these genomic changes must be associated with a profound difference in gene expression associated with iron and general metal metabolism. This was unmeasured in this study but is consistent with our previous results [33, 34]. Finally, our studies have important applications from the view of informing attempts to utilize metals such as gallium to control pathogenic and multidrug resistant bacteria. Despite optimism concerning the sustainability of metallic antimicrobials [4, 5, 24], bacteria can also rapidly evolve resistance to these materials as well.

## Supplementary Material

eoz025_Supplementary_Data
