# Supplementary material for: Experimental evolution of gallium resistance in Escherichia coli
Source: Evol Med Public Health. 2019 Sep 6;2019(1):169–80. doi: 10.1093/emph/eoz025 (PMC6928379; doi:10.1093/emph/eoz025)
Supplement: eoz025_Supplementary_Data [file eoz025_supplementary_data.zip › eoz025-Suppl_data/Supplemental Table 1.pdf]

Supplemental Table 1a. All Polymorphisms in Gallium-selected Populations

Position	Mutation	Gene	Ga1	Ga2	Ga3	Ga4	Ga5
87,023	A→G	ilvI →	0.000	0.000	0.000	0.055	0.000
90,720	C→A	rsmH →	0.000	0.000	0.000	0.066	0.000
433,518	C→A	ribD →	0.000	0.000	0.000	0.053	0.000
515,859	C→G	qmcA ← / → fetA	0.000	0.000	0.000	1.000	0.000
622,244	IS5 (–) +4 bp	fepD ← / → entS	0.351	0.000	0.639	0.000	0.000
1,234,632	A→G	nhaB ←	0.000	0.190	0.000	0.000	0.000
1,650,461	C→A	ydfE →	0.000	0.000	0.452	0.000	0.000
2,011,665	G→T	yedN ←	0.000	0.000	0.000	1.000	0.000
2,177,829	C→T	fbaB ←	0.000	0.000	0.000	0.053	0.000
2,519,222	T→G	yfeD → / ← gltX	0.000	0.000	0.000	0.058	0.000
2,519,228	C→A	yfeD → / ← gltX	0.000	0.000	0.000	0.059	0.000
2,629,042	IS5 (+) +4 bp	yfgF ← / → yfgG	0.000	0.000	0.500	0.000	0.000
2,636,200	C→A	der ←	0.000	0.000	0.000	0.075	0.000
2,785,563	G→A	ypjC ← / ← ileY	0.193	0.000	0.000	0.000	0.000
2,866,695	IS1 (+) +9 bp	rpoS ←	0.000	0.000	0.000	0.000	0.273
3,068,178	T→A	argO ←	0.000	0.000	0.000	0.056	0.000
3,287,319	C→T	agaI → / → yraH	0.161	0.000	0.000	0.000	0.000
3,375,962	C→T	dcuD →	0.000	0.000	0.000	0.053	0.000
3,448,200	T→C	rplN ← / ← rpsQ	0.000	0.000	0.000	0.052	0.000
3,651,029	IS2 (+) +5 bp	arsC → / → yhiS	0.000	0.000	0.000	1.000	0.000
3,664,265	C→G	gadW ←	0.000	0.000	0.000	0.060	0.000
3,815,810	Δ1 bp	pyrE ← / ← rph	0.000	0.000	0.000	0.080	0.000
3,815,895	Δ14 bp	rph ←	0.000	0.000	0.000	0.084	0.000
4,045,392	C→A	yihF →	0.000	0.000	0.000	0.061	0.000
4,105,639	C→A	cpxR ←	0.000	0.000	0.094	0.000	0.000
4,282,448	C→T	yjcF ←	0.000	0.000	0.000	0.051	0.000
4,349,066	T→A	dcuB ← / ← dcuR	0.147	0.000	0.000	0.000	0.000
4,507,739	G→T	insI1 ←	0.000	0.000	1.000	0.000	0.000
4,510,928	A→T	fecE ←	0.514	0.000	0.574	0.000	0.000
4,514,119	C→T	fecB ←	0.000	0.000	0.000	0.479	1.000
4,515,480	C→T	fecA ←	1.000	1.000	1.000	0.000	1.000
4,516,171	G→C	fecA ←	0.000	0.000	0.000	1.000	0.000
4,554,433	C→A	uxuB → / → uxuR	0.000	0.000	0.089	0.000	0.000
3,277,273:1	(TTCAACA)2→3	prlF →	0.184	0.000	0.000	0.000	0.000

Color coding: yellow, fixation; green, major variant; blue, minor variant

Supplemental Table 1b. Annotation of all Polymorphisms in Gallium-selected Populations

Gene	Annotation
ilvI →	E465G (GAG→GGG)
rsmH →	R209R (CGC→CGA)
ribD →	H22N (CAT→AAT)
qmcA ← / → fetA	intergenic (-86/-60)
fepD ← / → entS	intergenic (-55/-53)
nhaB ←	L29S (TTA→TCA)
ydfE →	pseudogene (384/765 nt)
yedN ←	pseudogene (376/678 nt)
fbaB ←	G246S (GGC→AGC)
yfeD → / ← gltX	intergenic (+17/+35)
yfeD → / ← gltX	intergenic (+23/+29)
yfgF ← / → yfgG	intergenic (-104/-245)
der ←	R386L (CGC→CTC)
ypjC ← / ← ileY	intergenic (-552/+199)
rpoS ←	coding (849-857/993 nt)
argO ←	S211C (AGT→TGT)
agaI → / → yraH	intergenic (+294/-107)
dcuD →	A365V (GCC→GTC)
rplN ← / ← rpsQ	intergenic (-51/+114)
arsC → / → yhiS	intergenic (+367/-258)
gadW ←	S118S (TCG→TCC)
pyrE ← / ← rph	intergenic (-42/+24)
rph ←	pseudogene (642-655/669 nt)
yihF →	V398V (GTC→GTA)
cpxR ←	R11L (CGA→CTA)
yjcF ←	A210T (GCT→ACT)
dcuB ← / ← dcuR	intergenic (-322/+249)
insI1 ←	D293E (GAC→GAA)
fecE ←	L177Q (CTG→CAG)
fecB ←	D64N (GAT→AAT)
fecA ←	G400S (GGC→AGC)
fecA ←	N169K (AAC→AAG)
uxuB → / → uxuR	intergenic (+72/-143)
prlF →	coding (272/336 nt)

Supplemental Table 1c. Description of all Polymorphisms in Gallium-selected Populations

Gene	Description
ilvI →	acetolactate synthase 3 large subunit
rsmH →	16S rRNA m(4)C1402 methyltransferase, SAM-dependent
ribD →	fused diaminohydroxyphosphoribosylaminopyrimidine[1]
qmcA ← / → fetA	PHB domain membrane-anchored putative protease/iron exporter,[2]
fepD ← / → entS	iron-enterobactin transporter subunit/enterobactin exporter, iron-regulated
nhaB ←	sodium:proton antiporter
ydfE →	Qin prophage; pseudogene;Phage or Prophage Related
yedN ←	pseudogene, IpaH/YopM family
fbaB ←	fructose-bisphosphate aldolase class I
yfeD → / ← gltX	DUF1323 family putative DNA-binding protein/glutamyl-tRNA synthetase
yfeD → / ← gltX	DUF1323 family putative DNA-binding protein/glutamyl-tRNA synthetase
yfgF ← / → yfgG	cyclic-di-GMP phosphodiesterase, anaerobic/uncharacterized protein
der ←	GTPase; multicopy suppressor of ftsJ
ypjC ← / ← ileY	pseudogene/tRNA-Ile
rpoS ←	RNA polymerase, sigma S (sigma 38) factor
argO ←	arginine transporter
agaI → / → yraH	galactosamine-6-phosphate isomerase/putative fimbrial-like adhesin protein
dcuD →	putative transporter
rplN ← / ← rpsQ	50S ribosomal subunit protein L14/30S ribosomal subunit protein S17
arsC → / → yhiS	arsenate reductase/pseudogene
gadW ←	transcriptional activator of gadA and gadBC; repressor of gadX
pyrE ← / ← rph	orotate phosphoribosyltransferase/ribonuclease PH (defective);enzyme;[3]
rph ←	ribonuclease PH (defective);enzyme; Degradation of RNA; RNase PH
yihF →	DUF945 family protein
cpxR ←	response regulator in two-component regulatory system with CpxA
yjcF ←	pentapeptide repeats protein
dcuB ← / ← dcuR	C4-dicarboxylate transporter, anaerobic; DcuS co-sensor/[4]
insI1 ←	IS30 transposase
fecE ←	iron-dicitrate transporter subunit
fecB ←	iron-dicitrate transporter subunit
fecA ←	ferric citrate outer membrane transporter
fecA ←	ferric citrate outer membrane transporter
uxuB → / → uxuR	D-mannonate oxidoreductase, NAD-dependent/[5]
prlF →	antitoxin of the SohA(PrlF)-YhaV toxin-antitoxin system

1. deaminase and 5-amino-6-(5-phosphoribosylamino) uracil reductase; 2.ATP-binding subunit, ABC transporter FetAB subunit; peroxide resistance protein; 3. Degradation of RNA; RNase PH; 4. response regulator in two-component regulatory system with DcuS; 5. fructuronate-inducible hexuronate regulon transcriptional repressor; autorepressor.
