# Supplementary material for: Experimental evolution of gallium resistance in Escherichia coli
Source: Evol Med Public Health. 2019 Sep 6;2019(1):169–80. doi: 10.1093/emph/eoz025 (PMC6928379; doi:10.1093/emph/eoz025)
Supplement: eoz025_Supplementary_Data [file eoz025_supplementary_data.zip › eoz025-Suppl_data/Supplemental Table 2.pdf]

Supplemental Table 2a. All Polymorphisms in Control Populations

Position	Gene	Mutation	C1	C2	C3	C4	C5
258,768	crl →	G→A	0.000	0.000	0.000	0.251	0.000
292,571	yagK ←	G→T	0.114	0.000	0.000	0.000	0.000
985,574	aspC ←	G→A	0.182	0.000	0.000	0.000	0.000
1,076,773	putA ←	C→A	0.000	0.000	0.000	0.082	0.000
1,362,050	puuR →	C→A	0.000	0.138	0.000	0.000	0.000
1,547,149	yddG ←	C→T	0.148	0.000	0.000	0.000	0.000
1,769,067	ydiJ ← / → ydiK	A→G	0.000	0.164	0.000	0.000	0.000
1,792,237	ydiV ← / ← nlpC	A→C	0.000	0.000	0.000	0.102	0.000
1,955,394	torZ ←	C→T	0.000	0.000	0.000	0.099	0.000
2,383,257	elaD →	Δ1 bp	0.108	0.000	0.000	0.000	0.000
2,712,451	trmN ←	A→G	0.000	0.000	0.000	0.102	0.000
2,988,528	yqeI →	C→A	0.000	0.222	0.000	0.000	0.000
3,034,615	lysS ←	C→T	0.121	0.000	0.000	0.000	0.000
3,504,511	frlR →	T→C	0.129	0.000	0.000	0.000	0.000
3,651,029	arsC → / → yhiS	IS2 (+) +5 bp	0.000	0.000	0.000	0.938	0.000
3,983,887	aslB →	G→A	0.000	0.000	0.000	0.095	0.000
3,991,328	cyaA →	A→T	0.000	0.000	0.000	0.106	0.000
4,097,105	rhaB ←	(TGT)3→2	0.000	0.000	0.000	0.103	0.000
4,160,216	sthA ←	G→A	0.129	0.000	0.000	0.000	0.000
4,182,820	rpoB →	C→T	0.000	0.000	0.000	0.338	0.000
4,183,378	rpoB →	T→C	0.320	0.655	0.381	0.000	0.000
4,183,379	rpoB →	C→T	0.000	0.000	0.000	0.607	0.000
4,186,532	rpoC →	A→G	0.236	0.000	0.000	0.000	0.000
4,479,020	rraB → / ← yjgM	A→T	0.000	0.000	0.216	0.000	0.000
4,496,351	ahr ← / → leuX	C→T	0.000	0.000	0.149	0.000	0.000

Color coding: yellow, fixation; green, major variant; blue, minor variant

Supplemental Table 2b. Annotation of all Polymorphisms in Control Populations

Gene	Annotation
crl →	pseudogene (93/331 nt)
yagK ←	Y126* (TAC→TAA)
aspC ←	L46L (CTG→TTG)
putA ←	A704S (GCT→TCT)
puuR →	P47H (CCT→CAT)
yddG ←	T7T (ACG→ACA)
ydiJ ← / → ydiK	intergenic (-382/-7)
ydiV ← / ← nlpC	intergenic (-217/+30)
torZ ←	A538A (GCG→GCA)
elaD →	coding (545/1212 nt)
trmN ←	I105T (ATT→ACT)
yqeI →	I9I (ATC→ATA)
lysS ←	R187H (CGC→CAC)
frlR →	Y154H (TAT→CAT)
arsC → / → yhiS	intergenic (+367/-258)
aslB →	M310I (ATG→ATA)
cyaA →	K59I (AAA→ATA)
rhaB ←	coding (342-344/1470 nt)
sthA ←	A192V (GCG→GTG)
rpoB →	H526Y (CAC→TAC)
rpoB →	S712P (TCC→CCC)
rpoB →	S712F (TCC→TTC)
rpoC →	K395E (AAA→GAA)
rraB → / ← yjgM	intergenic (+131/+14)
ahr ← / → leuX	intergenic (-142/-54)

Supplemental Table 2c. Description of all Polymorphisms in Control Populations

Gene	Description
crl →	pseudogene, sigma factor-binding protein, stimulates RNA polymerase holoenzyme[1]
yagK ←	CP4-6 prophage; conserved protein
aspC ←	aspartate aminotransferase, PLP-dependent
putA ←	fused DNA-binding transcriptional regulator/proline dehydrogenase/[2]
puuR →	repressor for the divergent puu operons, putrescine inducible
yddG ←	aromatic amino acid exporter
ydiJ ← / → ydiK	putative FAD-linked oxidoreductase/UPF0118 family inner membrane protein
ydiV ← / ← nlpC	anti-FlhD4C2 factor, inactive EAL family phosphodiesterase/[3]
torZ ←	trimethylamine N-oxide reductase system III, catalytic subunit
elaD →	protease, capable of cleaving an AMC-ubiquitin model substrate
trmN ←	tRNA1(Val) (adenine(37)-N6)-methyltransferase
yqeI →	putative transcriptional regulator
lysS ←	lysine tRNA synthetase, constitutive
frlR →	putative DNA-binding transcriptional regulator
arsC → / → yhiS	arsenate reductase/pseudogene
aslB →	putative AslA-specific sulfatase-maturating enzyme
cyaA →	adenylate cyclase
rhaB ←	rhamnulokinase
sthA ←	pyridine nucleotide transhydrogenase, soluble
rpoB →	RNA polymerase, beta subunit
rpoC →	RNA polymerase, beta prime subunit
rraB → / ← yjgM	protein inhibitor of RNase E/putative acetyltransferase
ahr ← / → leuX	aldehyde reductase, NADPH-dependent, Zn-containing, broad specificity/tRNA-Leu

1. formation; regulator Surface structures; transcriptional regulator of cryptic csgA gene for curli surface fibers.
2. pyrroline-5-carboxylate dehydrogenase
3. putative C40 clan peptidase lipoprotein
